# Dissolved Oxygen Decline in Northern Beibu Gulf Summer Bottom Waters: Reserve Management Insights from Microbiome Analysis

**DOI:** 10.3390/microorganisms13081945

**Published:** 2025-08-20

**Authors:** Chunyan Peng, Ying Liu, Yuyue Qin, Dan Sun, Jixin Jia, Zongsheng Xie, Bin Gong

**Affiliations:** The Guangxi Key Laboratory of Beibu Gulf Marine Biodiversity Conservation, College of Marine Sciences, Beibu Gulf University, Qinzhou 535011, China

**Keywords:** coastal ecosystems, microbiota, dissolved oxygen, co-occurrence network, PLS-PM

## Abstract

The Sanniang Bay (SNB) and Dafeng River Estuary (DFR) in the Northern Beibu Gulf, China, are critical habitats for the Indo-Pacific humpback dolphin (*Sousa chinensis*). However, whether and how the decreased dissolved oxygen (DO) has happened in bottom seawater remains poorly understood. This study investigated DO depletion and microbial community responses using a multidisciplinary approach. High-resolution spatiotemporal sampling (16 stations across four seasons) was combined with functional annotation of prokaryotic taxa (FAPROTAX) to characterize anaerobic metabolic pathways and quantitative PCR (qPCR) targeting *dsrA* and *dsrB* genes to quantify sulfate-reducing bacteria. Partial least-squares path modeling (PLS-PM) was employed to statistically link environmental variables (seawater properties and nutrients) to microbial community structure. Results revealed pronounced bottom DO declining to 5.44 and 7.09 mg L^−1^, a level approaching sub-optimal state (4.0–4.8 mg L^−1^) in September. Elevated chlorophyll-a (Chl-a) near the SDH coincided with anaerobic microbial enrichment, including sulfate reducers (*dsrA*/*dsrB* abundance: SNB > DFR). PLS-PM identified seawater properties (turbidity, DO, pH) and nitrogen as key drivers of anaerobic taxa distribution. Co-occurrence network analysis further demonstrated distinct microbial modules in SNB (phytoplankton-associated denitrifiers) and DFR (autotrophic sulfur oxidizers, nitrogen fixation, and denitrification). These findings highlight how environmental factors drive decreased DO, reshaping microbial networks and threatening coastal ecosystems. This work underscores the need for regulating aquaculture/agricultural runoff to limit eutrophication-driven hypoxia and temporarily restrict human activities in SNB during peak hypoxia (September–October).

## 1. Introduction

In marine environments, summer stratification and hypoxic (DO concentrations below 2 mg L^−1^)) in bottom seawater have been reported previously [1]. Some well-known hypoxic coastal regions include the northern Gulf of Mexico [2], the Bohai Sea [3], the Gulf of Trieste [4], the Changjiang Estuary [5], and the Seto Inland Sea [6]. In most cases, the mechanism behind the hypoxia in bottom seawater has been correlated with eutrophication, which involves vast riverine loading of nitrogen (N) and phosphorus (P) into the estuary and phytoplankton blooms during spring in the surface water, subsequently causing phytoplankton to sink to the bottom [7]. With summer stratification, when the downward flux of phytoplankton brings extensive organic matter to the bottom, oxygen (O_2_) is consumed rapidly by respiration [8]. If the O_2_ consumption during respiration exceeds the supply of oxygen-rich waters, then O_2_ depletion in the bottom seawater will occur [9]. In brief, eutrophication and poor hydrodynamic conditions—low vertical mixing rates and restricted horizontal water exchange—are two key critical factors that determine the occurrence of hypoxia. However, in some coastal regions, where the hydrodynamic conditions have been significantly weakened, whether the decreased DO (referring to conditions where DO levels are above hypoxia threshold but show a significant decline relative to baseline or surface water concentrations) has occurred and its impact on microbial communities requires further investigation.

The Northern Beibu Gulf, a biodiverse coastal region in China, harbors critical habitats for the Indo-Pacific humpback dolphin (*Sousa chinensis*), notably in Sanniang Bay (SNB) and the Dafeng River Estuary (DFR) [10]. However, large-scale infrastructure projects, such as the Sandun Marine Highway (SDH) and adjacent land reclamation initiated in 2010, have dramatically altered this ecosystem [11]. The SDH’s construction fragmented natural hydrodynamic pathways, reducing water circulation and increasing sedimentation rates in SNB [12]. These changes promote the accumulation of fine-grained, organic-rich sediments—a process exacerbated by seasonal thermal stratification in summer [13]. Stratification isolates bottom waters, limiting oxygen replenishment and creating anaerobic conditions conducive to microbial communities that thrive in low-oxygen environments [14].

Sulfate-reducing bacteria (SRB) (identified via *dsrA*/*dsrB* gene quantification) metabolize organic carbon using sulfate as a terminal electron acceptor [15], producing hydrogen sulfide (H_2_S)—a toxin that acidifies sediments and indicates anaerobic or low DO [9]. These microbes also mediate critical biogeochemical cycles (e.g., sulfur, nitrogen), destabilizing nutrient fluxes and promoting hypoxic conditions that cascade through the food web. For instance, SRB-dominated sediments correlate with declines in benthic macrofauna (e.g., crustaceans, mollusks), key prey for humpback dolphins [16]. Anaerobic microbes are not merely passive indicators of hypoxia but active drivers of ecosystem degradation. Their metabolic byproducts (e.g., H_2_S, methane) create feedback loops that sustain low-oxygen conditions, rendering habitats unsuitable for aerobic species. This microbial transformation is particularly consequential in SNB, where black, sulfidic sediments coincide with reduced dolphin sightings and shifts in their core distribution to the DFR [10]. We hypothesize that infrastructure-induced hydrodynamics have altered microbial communities of seawater—with those proximate to SDH tending toward anaerobiosis—and thereby driven shifts in apex predator ecology near such areas. This study integrates functional annotation of prokaryotic taxa (FAPROTAX), qPCR of *dsrA*/*dsrB* genes, and partial least-squares path modeling (PLS-PM) to (1) quantify spatiotemporal variations in DO across SDH-impacted zones in four different seasons, (2) establish how decreased DO shapes microbial communities in the SNB-SDH area, and (3) evaluate the cascading effects of microbial-mediated hypoxia on humpback dolphin habitat quality.

## 2. Materials and Methods

### 2.1. Study Site

**Figure 1:**
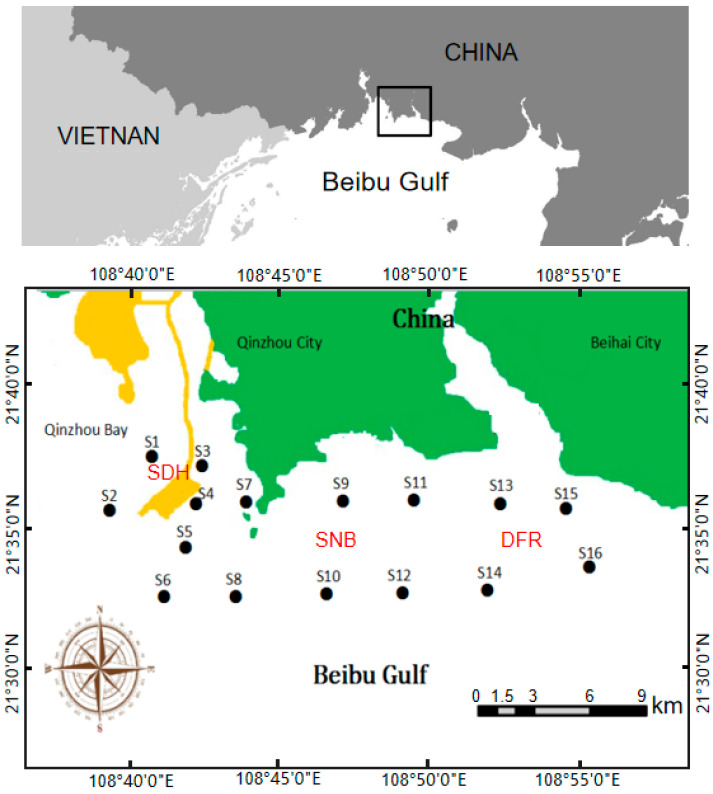
Study region contains the coastal area of the North Beibu Gulf, China. SDH, Sandun Marine Highway; SNB, Sanniang Bay; DFR, Dafengjiang River Estuary.

The study region is located in the coastal area of the North Beibu Gulf, China. Studies were conducted in Qinzhou Bay, the SDH, Sanniang Bay (SNB), and the DFR (21.63° N, 108.69° E–21.56° N, 108.93° E) (Figure 1). This region has attracted broad attention because it is one of the eight known habitats for humpback dolphin subpopulations [17]. From the 1990s to 2010s, large-scale human activities intensely changed the morphological characteristics of Qinzhou Bay [11]. Among coastal land reclamation projects, the SDH has been in the process of being constructed since approximately 2010 (Figure 1). The SDH significantly varied the hydrodynamic conditions in Qinzhou Bay [12]. Major impacts on the sediment grain size [12] and distribution patterns of humpback dolphins [16] in this area have been investigated. In our previous work, sediments from the DFR were found to be sandy and oligotrophic, while sediments in SNB were found to be black, muddy, and anaerobic [14]. Compared to the results of a previous survey, the humpback dolphin core distribution zone appears to have slightly changed from “SNB and the DFR” to the DFR [16].

To ensure spatial representativeness, 16 sampling stations (S1–S16, depth ranged from 2.3–10.7 m) were strategically distributed across the study area (Figure 1 and Figure S1), covering gradients of hydrodynamic influence from the SDH to the DFR. Stations were categorized into two groups: SNB (Stations S1–S10, positioned in areas with historically high humpback dolphin sightings and sediment deposition zones altered by SDH construction) and DFR (Stations S11–S16, located near freshwater inflow points to capture mixing dynamics and serve as a less-impacted reference region). Stations were spaced 1–2 km apart to resolve fine-scale spatial variability, with placement informed by bathymetric surveys and prior habitat utilization studies [16]. The bathymetric data of the sampling sites were illustrated in Figure S1.

### 2.2. Sampling and Physicochemical Analysis

Seawater samples were collected during March (Mar), July (Jul), September (Sep), and December (Dec) from 16 stations (S1–S16, Figure 1). Stations S1–S10 were grouped as SNB, while S11–S16 were grouped as the DFR. GPS position and oceanographic characteristics were measured at each station. Continuous profiles of salinity, temperature, and depth were detected with Midas conductivity–temperature–depth (CTD) sensors (Valeport, UK) [18]. Seawater was taken from the surface, middle, and bottom of the station areas using Niskin bottles (10 L, KC-Denmark, Silkeborg, Denmark) according to the depth of the station’s seawater. The bacterial community, nutrients (total nitrogen (TN), nitrate (NO_3_^−^), nitrite (NO_2_^−^), ammonium ion (NH_4_^+^), total phosphorus (TP), and phosphate radical (PO_4_^3−^)), and chlorophyll-a (Chl-a) were measured. One-liter seawater samples were immediately filtered through 0.22 μm polycarbonate filters (Millipore, MA, USA) and transported to the laboratory [19]. Filters were stored at −80 °C for bacterial community analyses. The water passing through filters was immediately used for nutrient analysis. Nutrient concentrations (NO_3_^−^, NO_2_^−^, NH_4_^+^, PO_4_^3−^, TP, and TN) were determined with a continuous flow analyzer (QuAAtro, SEAL, Germany) according to the methods described previously [20] within 24 h. The analytical error for each nutrient element was less than 0.03 µM. The DO concentration of the seawater from the surface, middle area, and bottom was determined by using a high-accuracy Winkler titration method [21]. Then, 2.0–3.0 L of seawater for Chl-a determination was filtered through GF/F filters, and the filters were frozen at −20 °C. In addition, the Chl-a on the filters was extracted with 90% acetone and measured with the method described by Ghaemi [22].

All physicochemical and biological measurements were conducted in triplicate to ensure precision. Triplicate aliquots from each Niskin bottle were analyzed independently, and results were averaged to minimize pipetting and instrument error. Triplicate GF/F filters per station were processed, with coefficients of variation (CV) < 5% deemed acceptable. Spatial distributions of environmental variables (e.g., temperature, salinity, Chl-a, nutrients) were interpolated using ordinary kriging in ArcGIS Pro 3.0.2 [23], with the following parameters: Spherical Model for temperature and salinity, Exponential Model for NO_3_^−^, NH_4_^+^, Chl-a, and depth; Nugget, 0.1 (representing measurement error); Sill, 1.2 (total variance); Range, 10–15 km for salinity, depth, and temperature, 5–8 km for NO_3_^−^, NH_4_^+^, and Chl-a.

### 2.3. DNA Extraction, PCR, and Amplicon Sequencing 16S rRNA Gene

The DNA of the bacterial cells on the filters was extracted with the AquaScreen^®^ Fast Extraction kit (Minerva BioLabs, Berlin, Germany) following the manufacturer’s instructions [24]. After detection by a NanoDrop spectrophotometer (ThermoFisher Scientific, MA, USA) and agarose gel electrophoresis [25], the extracted DNA was used to amplify the V5–V7 region of the 16S rRNA gene (FW(799F): AACMGGATTAGATACCCKG; RV(1193R): ACGTCATCCCCACCTTCC) [26]. Amplicon sequencing was conducted with the Illumina HiSeq2500 platform of Majorbio Bio-pharm Technology Company (Shanghai, China) [27]. The raw data were uploaded into the online data repository GenBank with accession number PRJNA924999.

### 2.4. The qPCR Analysis of Sulfate-Reducing Genes dsrA and dsrB

The quantitative PCR (qPCR) was used to determine the abundance of genes *dsrA* and *dsrB* [15]. The qPCR analysis was conducted using the real-time fluorescent quantitative PCR instrument (CFX96 TOUCH, Bio-Rad, Hercules, CA, USA). The total DNA extracted from bacteria on filters was used as template. The primers of genes *dsrA* and *dsrB* are listed as follows: dsrA-F-ACSCACTGGAAGCACG, dsrA-R-GGTGGAGCCGTGCATGTT; dsrB-F-CAACATCGTYCAYACCCAGGG, dsrB-R-GTGTAGCAGTTACCGCA [28]. The PCR procedure was as follows: denaturation (94 °C for 9 min), denaturation (40 cycles at 94 °C for 30 s), annealing (55 °C for 30 s), and extension (72 °C for 45 s) [29]. All the qPCR reactions were conducted in triplicate. The relative abundance of genes *dsrA* and *dsrB* was calculated using the 16S RNA gene as a reference [28].

### 2.5. Bioinformatics

The raw data of 16S rRNA gene sequences were processed by quality trimming the reads [30], merging the paired-end reads [31], and removing the chimeric sequences [32]. Then, a QIIME 2 plugin, q2-feature-classifier, was used to cluster the sequences into operational taxonomic units (OTUs) [33]. The SINTAX classifier was used for taxonomy annotations against the Silva 138 Database [34]. A total of 1.3 Gb of sequences were generated from 64 samples, with an average of 71,397 clean reads per sample and an average length of 307 bp. The OTU functional information was annotated against the functional annotation of prokaryotic taxa (FAPROTAX) database [35]. The OTUs annotated with certain functional profiles involved in the biogeochemical cycles of C, N, and S were calculated, and this number was used to determine the relative abundance of functional microbial groups at each sampling site [36]. The spatial distribution of the relative abundance of the functional microbiota at the 16 sampling sites was interpolated using the kriging method [23]. Partial least-squares path modeling (PLS-PM) analysis was conducted using the “plspm” package in R (version 0.5.1) with 1000 bootstraps to assess path coefficients [37].

### 2.6. Statistical Analysis

The sample size (n = 64 samples across 16 stations × 4 seasons) provided adequate power to detect significant differences in microbial community structure and environmental variables. ANOVA/Welch’s *t*-test (using IBM^®^ SPSS Statistics, version 28.0) was applied after confirming homogeneity of variances (Levene’s test, *p* < 0.05).

## 3. Results

### 3.1. Seasonal Variation of Environmental Variables

**Figure 2:**
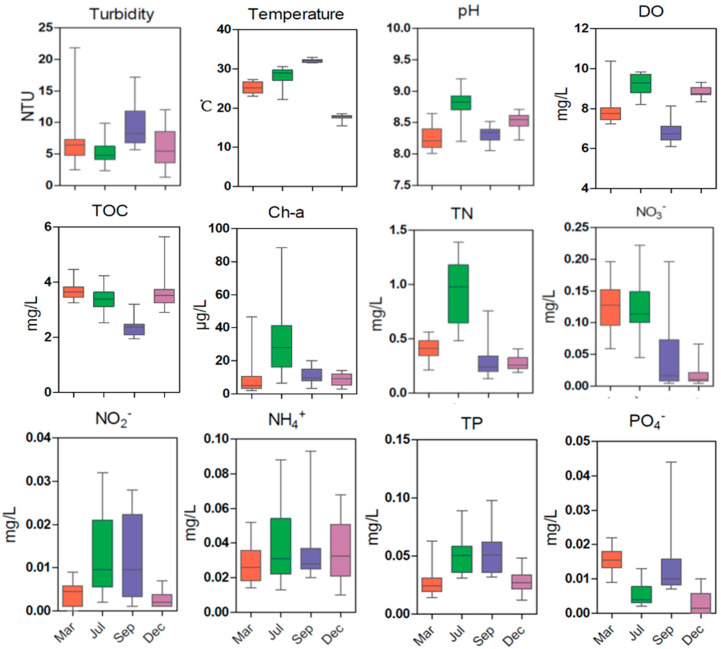
Environmental variables of seawater measured in four seasons (March (Mar), July (Jul), September (Sep), and December (Dec)) in the SNB and DFR regions in the Beibu Gulf. The measured physicochemical properties of the seawater include turbidity, temperature, pH, DO, TOC, Chl-a, TN, NO_3_^−^, NO_2_^−^, NH_4_^+^, TP, and PO_4_^−^. Y-axes represent the concentrations of the environmental variables. DO: dissolved oxygen; TOC: total organic carbon; Chl-a: chlorophyll; TN: total nitrogen; NO_3_^−^: nitrate; NO_2_^−^: nitrite; NH_4_^+^: ammonium ion; TP: total phosphorus; PO_4_^3−^: phosphate radical. ANOVA test was applied for the statistical analysis, and error bar is SD (n = 16).

The seasonal variations in environmental characteristics are illustrated in Figure 2. Temperatures in March were approximately 25.2 °C, increasing to 28.1 °C in July and reaching a maximum of over 32 °C in September, until they decreased again in December to below 18 °C. The seasonal change in pH was generally consistent with that in DO, with increasing trends from March to July, reaching similar peaks in July, then decreasing to low values in September, and finally increasing in December. Interestingly, PO_4_^3−^ exhibited the opposite seasonal fluctuation trend from that of pH and DO. The highest concentration of turbidity was observed in September, whereas the total organic carbon (TOC) had the opposite trend, with the lowest value detected in September. The Chl-a and TN varied following a similar pattern, with the maximum concentrations in July. Higher average concentrations of NO_3_^−^ appeared in March and July, ranging between 0.125 and 0.127 mg L^−1^. We observed higher average concentrations of NO_2_^−^ in July and September, which varied widely, ranging between 0.0116 and 0.0126 mg L^−1^. The seasonal average concentrations of TP exhibited similar trends to those of NO_2_^−^ throughout the year.

### 3.2. Spatial Variation in Oceanographic Characteristics, Nutrients, and Chl-a

Nutrient profiles (TN, NO_3_^−^, NO_2_^−^, NH_4_^+^, TP, and PO_4_^3−^) were characterized by an obvious increase in NO_3_^−^ and NH_4_^+^ concentrations in the estuary of the DFR in September. In March and December, some other high-NO_3_^−^ (or NH_4_^+^) areas were accidentally found in the SNB area, which is a regional tourist attraction. The variation in Chl-a had significant seasonal and regional fluctuations. From March to July, the highest Chl-a concentrations appeared in the estuary of the DFR. However, Chl-a near the SDH was higher than that in the DFR in September. In winter (December), there was a substantial temperature decrease (13–15 °C), and a higher Chl-a belt appeared along the coastline off the SNB region. Unlike nutrients and Chl-a, seasonal variation showed a distinctly regional signal. There was a less clear seasonal cycle for temperature and salinity (Figure S2).

### 3.3. Seasonal Variation in Dissolved Oxygen (DO) in the SNB and DFR Regions

**Figure 3:**
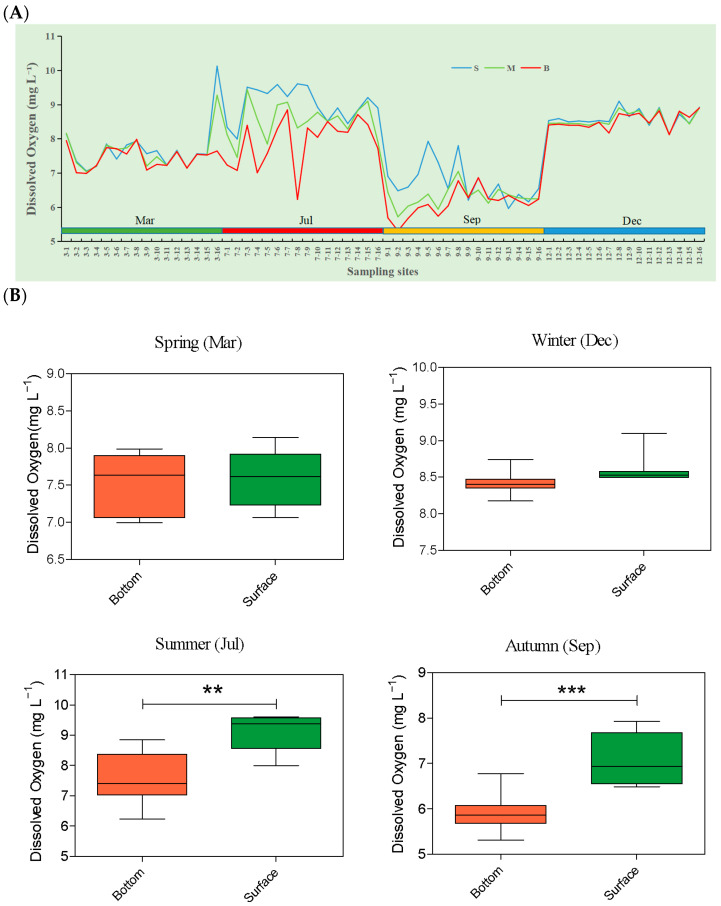
Dissolved oxygen (DO) in the seawater in the SNB and DFR regions in the Beibu Gulf. (**A**) Seasonal variation in DO of the bottom water (B), middle water (M), and surface water (S) during March (Mar), July (Jul), September (Sep), and December (Dec) at different sampling sites; (**B**) Comparison of surface and bottom DO in different seasons. Asterisks indicate the significance of the *t*-test analysis (** *p* < 0.01; *** *p* < 0.001).

The DO in the SNB and DFR regions experienced significant seasonal fluctuations. The DO in July (average seawater temperature: 28.1 °C) was the highest year-round, whereas a strong decrease appeared in September (average seawater temperature: 32.1 °C) (Figure 3A). A high-temperature-induced stratification of seawater was observed in summer (July and September). Vertical decreases in DO from the surface to the bottom were observed in July and September (Figure 3A). In July, in the SNB region (stations S1–S10), surface DO was significantly higher, ranging between 8.19 and 9.84 mg L^−1^, than bottom DO, ranging between 6.18 and 8.60 mg L^−1^ (Welch’s *t*-test: t = 4.11, df = 12, *p* < 0.001, 95% CI [−2.37, −0.73]). In September, bottom DO (5.44 and 7.09 mg L^−1^) was significantly lower than surface DO (6.70 and 8.12 mg L^−1^) (Welch’s *t*-test: t = 4.60, df = 14, *p* < 0.001, 95% CI [−1.69, −0.62]), indicating a large-magnitude decline (Figure 3B).

### 3.4. Spatial Distribution of Functional Bacterial Groups in September When Seawater Stratification and a DO Decrease Occurred in the SNB Region

**Figure 4:**
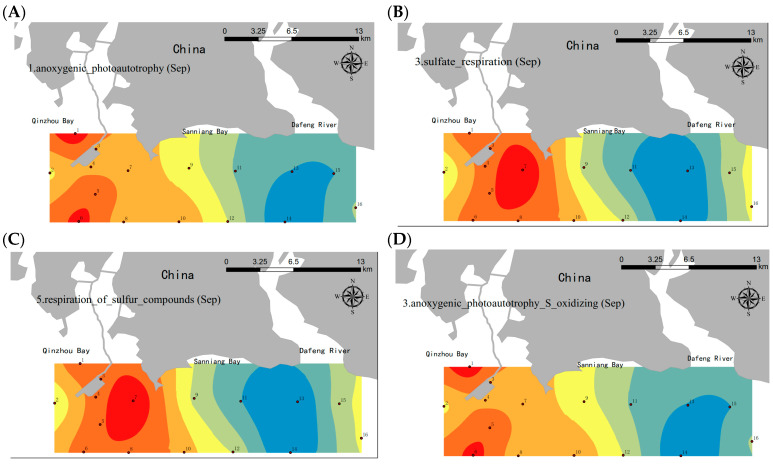

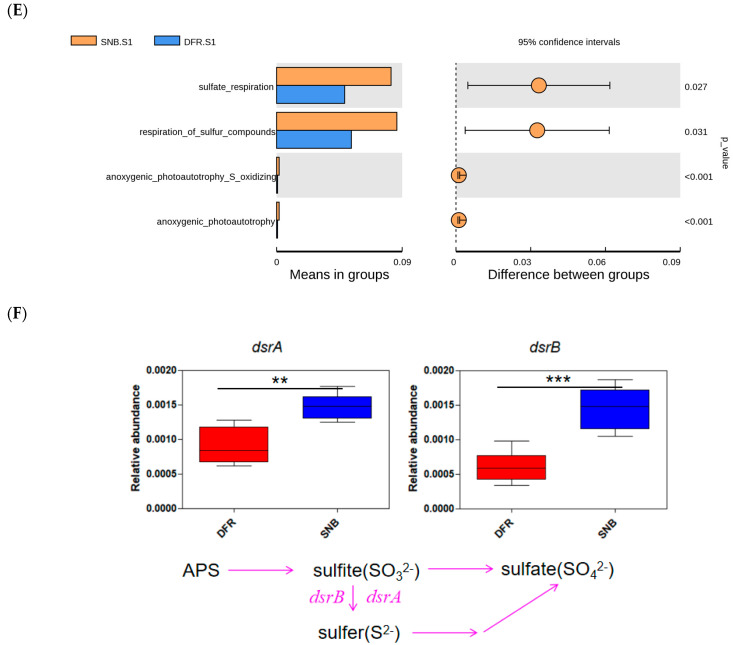
Spatial distribution of functional bacterial groups in the coastal regions of SNB and the DFR in September. Functional prediction of bacterial communities was conducted using the functional annotation of prokaryotic taxa (FAPROTAX) method. The relative abundance of the bacteria involved in certain metabolic functions is demonstrated on the map using ArcGIS by the kriging method. Functional bacterial groups include (**A**) anoxygenic photoautotrophic; (**B**) sulfate respiration; (**C**) respiration of sulfur compounds; (**D**) anoxygenic photoautotrophic S-oxidation. The blue to red colors indicate lower to higher concentrations, respectively. (**E**) The different abundances of the functional groups in SNB and the DFR were illustrated by a *t*-test; (**F**) The qPCR analysis of sulfate-reducing genes *dsrA* and *dsrB*. Asterisks indicate the significance of the *t*-test analysis (** *p* < 0.01; *** *p* < 0.001).

FAPROTAX analysis was utilized to compare the functional microbial groups between the SNB and DFR regions. There were fewer clear characteristics and spatial distributions of functional bacterial groups throughout the four seasons, except for September (Figure 4). In September, the proportion of microorganisms involved in sulfate respiration and the respiration of sulfur compounds in SNB was significantly higher than that in the DFR (Welch’s *t*-test, *p* < 0.05) (Figure 4B,E). In addition, a relatively higher abundance of anoxygenic photoautotrophic and anoxygenic photoautotrophic S-oxidation OTUs was detected in SNB than in the DFR (Welch’s *t*-test, *p* < 0.001) (Figure 4B,E). The qPCR revealed significantly higher abundance of sulfate-reducing bacteria (SRB) genes (*dsrA*/*dsrB*) in SNB vs. DFR (ANOVA: F_1,30_ = 18.7, *p* < 0.01, Figure 4F). This indicates intensified anaerobic metabolism driven by organic matter degradation under low-DO conditions, consistent with field observations of blackened, sulfidic sediments in SNB [14]. SRB proliferation directly threatens benthic ecosystems through hydrogen sulfide (H_2_S) production—a neurotoxin linked to macrofaunal mortality [2] and prey depletion for humpback dolphins.

**Figure 5:**
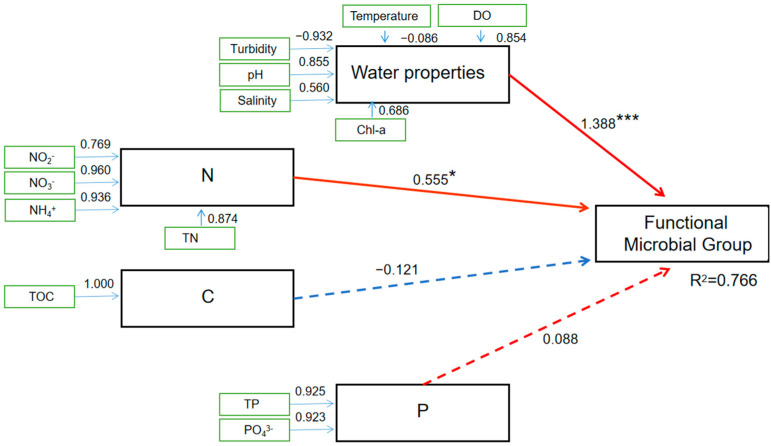
PLS-PM analysis illustrating the relationship between environmental characteristics (seawater properties, N, C, and P) and the spatial distribution of functional bacterial communities (anoxygenic photoautotrophic, sulfite respiration, respiration of sulfur compounds, and anoxygenic photoautotrophic S-oxidation) in the coastal regions of SNB and the DFR. The red arrow indicates a positive relationship (increasing environmental elements will increase the abundance of algae), while the blue arrow indicates a negative correlation (increasing environmental elements will reduce the abundance of algae). The solid arrow indicates a significant relationship (*, *Pr* < 0.05; ***, *Pr* < 0.001), while the dotted arrow indicates a nonsignificant relationship (*Pr* > 0.05).

PLS-PM was utilized to analyze the contributions of environmental factors (seawater properties, N, C, and P) to the spatial distribution of functional microbial groups in the coastal regions of SNB and the DFR (Figure 5). The PLS-PM analysis indicated a good match of our data according to the indices of model fit (R^2^ = 0.766). The PLS-PM analysis also showed that seawater properties (temperature, DO, pH, turbidity, salinity, and Chl-a), nitrogen (N), carbon (C), and phosphorus (P) had positive or negative impacts on the OTU numbers distributed at the sampling sites. Of the factors, seawater properties were the most influential factor positively impacting the distribution of the functional microbial groups (Estimate = 1.388, Std. Error = 0.412, *t* value = 3.37, *Pr* < 0.001), followed by N (Estimate = 0.555, Std. Error = 0.250, *t* value = 2.22, *Pr* < 0.05). P and C exhibited positive and negative impacts, respectively. However, none of these impacts were found to be significant (Figure 5).

### 3.5. Effect of Environmental Characteristics on the Co-Occurrence Network in September When Seawater Stratification and a DO Decrease Occurred in the SNB Region

**Figure 6:**
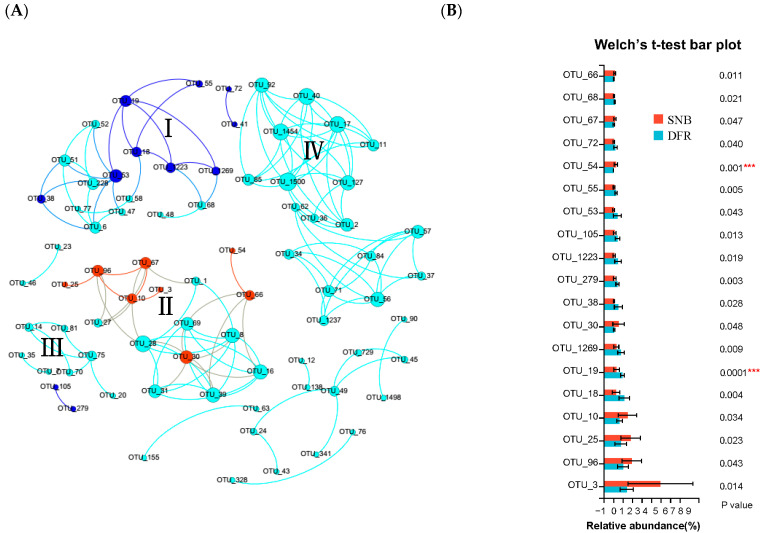
Co-occurrence network constructed to indicate interactions among different OTUs in bacterial communities in the coastal regions of SNB and the DFR. (**A**) Modules I–IV. All OTUs present in the co-occurrence network were subjected to abundance analysis, and the OTUs with different abundances in SNB (red bar) and the DFR (blue bar) in the co-occurrence network are illustrated by Welch’s *t*-test (*p* < 0.05) in (**B**). The OTUs enriched in SNB were indicated as red nodes (belonging to module II), and OTUs enriched in the DFR were shown as deep blue nodes (belonging to module I) in (**A**). Asterisks indicate the significance of the *t*-test analysis (*** *p* < 0.001).

**Figure 7:**
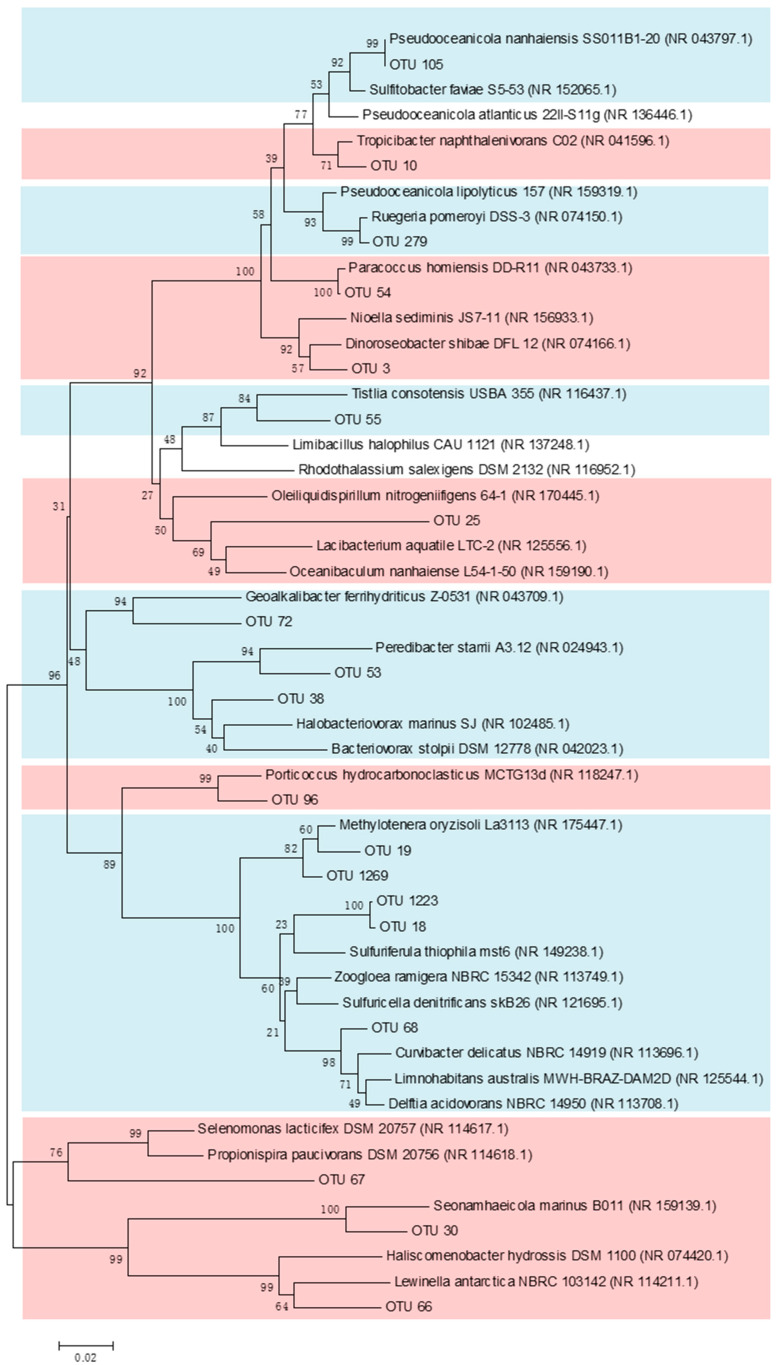
Phylogenetic tree constructed using sequences of OTUs significantly enriched in SNB or the DFR in the co-occurrence network (Figure 6) and their most related reference sequences. Numbers following the Latin names of the reference sequences are GenBank accessions. The OTUs in the branches marked with red (OTU10, OTU54, OTU3, OTU25, OTU96, OTU67, OTU30, OTU66) are significantly enriched in SNB, while OTUs marked with blue (OTU105, OTU279, OTU55, OTU72, OTU53, OTU38, OTU19, OTU18, OTU68) are significantly enriched in the DFR.

A co-occurrence network was constructed to determine the interactions among the different OTUs in the bacterial communities in the coastal regions of SNB and the DFR (Figure 6). As indicated in Figure 6, 77 OTUs (nodes) remained in the co-occurrence network using the data of the bacterial communities in September (|Coefficient| > 0.8, *p*-value < 0.05). All of the OTUs in the network were positively correlated. The nodes belonged to four phyla (Proteobacteria, Actinobacteria, Bacteroidetes, and Marinimicrobia). Four modules (modules I to IV, a module defined as a group of OTUs connected more tightly among themselves but connected loosely to the OTUs of the modules) that possessed over five nodes were recovered from the network. From the network, we found that 19 OTUs were significantly enriched in SNB or the DFR (Welch’s *t*-test, *p* < 0.05) (Figure 6). More interestingly, 9 OTUs belonging to module I, which were enriched in the DFR, were identified in the network (Figure 6). From the phylogenetic tree, we found that OTUs enriched in the DFR were most related to *Pseudooceanicola*, *Ruegeria*, *Tistlia*, *Geoalkalibacter*, *Peredibacter*, *Halobacteriovorax*, *Methylotenera*, *Sulfuriferula*, and *Curvibacter* (Figure 7). Similarly, 8 OTUs belonging to module II, which were enriched in SNB, were identified in the network (Figure 7). These OTUs were found to be most related to *Tropicibacter*, *Paracoccus*, *Dinoroseobacter*, *Oceanibaculum*, *Porticoccus*, *Propionispira*, *Seonamhaeicola*, and *Lewinella*.

**Figure 8:**
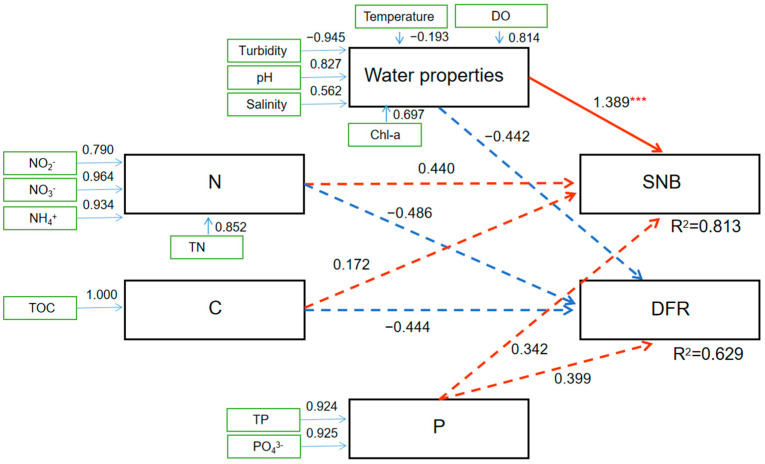
PLS-PM analysis illustrating the relationship between environmental characteristics (seawater properties, N, C, and P) and OTUs specifically enriched in SNB or the DFR in the co-occurrence network. Red arrows indicate a positive relationship (the increase in the value will increase the abundance of OTUs), while blue arrows indicate a negative correlation (the increasing value will reduce the abundance of OTUs). Solid arrows indicate a significant relationship (***, *Pr* < 0.001), while dotted arrows indicate a nonsignificant relationship (*Pr* > 0.05).

To understand how environmental factors impact the co-occurrence network, we conducted path modeling to analyze the contributions of environmental elements (seawater properties, N, C, and P) to the abundance of OTUs specifically enriched in SNB or the DFR in the co-occurrence network (Figure 8). The PLS-PM analysis illustrated that seawater properties (temperature, DO, pH, turbidity, salinity, and Chl-a), N, C, and P had positive impacts on the OTUs enriched in SNB. However, seawater properties, N, and C exhibited negative impacts on the OTUs enriched in the DFR. Only seawater properties were the most influential factors positively impacting the abundance of OTUs specifically enriched in SNB (Estimate = 1.40, Std. Error = 0.396, *t* value = 3.53, *Pr* < 0.001) (Figure 8).

## 4. Discussion

### 4.1. Lower Dissolved Oxygen (DO) Profile in the Bottom Seawater of the Sanniang Bay (SNB) Region

In this work, a strong decrease in DO appeared in the bottom seawater of SNB in September when the average temperature of seawater was the highest (32.1 °C). Vertical decreases in DO from the surface to the bottom were observed in July and September. Specifically, in September, bottom DO showed a pronounced decrease compared with surface DO in SNB. Compared to our previous publication [14], this work provides direct evidence that summer DO decreases specifically in the bottom layer of the SNB region, and this bottom water oxygen reduction trend is harmful to marine ecosystems [38]. When the DO falls between 71.9 and 150 µmol kg^−1^, a chronic hypoxia condition, chronic effects may occur under prolonged exposure [39]. In our work, the bottom almost reached the threshold of chronic hypoxia, and some sampling sites had values close to 150 µmol kg^−1^. This result may partially explain our previous finding that habitat utilization of SNB by the humpback dolphin has changed since the construction of the SDH [16]. This change also occurred over a time when crustaceans and mollusks at the bottom of the SNB substantially decreased [14].

The decrease in DO that appeared in the bottom seawater of SNB in September may be correlated with the construction of the SDH and artificial island near this region. The SDH and artificial island have been under construction since 2010 [11], and planned land reclamation will cover 79 km^2^ by 2025 [11]. Moreover, due to land reclamation, sediment grain size clearly differed throughout the study site, and the material sources and hydrodynamic conditions also varied [12]. In our previous study, we carried out an investigation on environmental characteristics and conducted a microbiome analysis from the SDH to DFR area [14]. Comparably higher concentrations of NH_4_^+^-N, NO_3_^−^-N, dissolved reactive phosphorus (DRP), Pb, and Cd were detected in SNB than in the DFR [14]. Notably, some anaerobic microbiota were enriched in the sediments of SNB, and muddy black soil at the bottom of SNB was further discovered [14]. Therefore, the previous results are in good agreement with those of this work. An inclination toward hypoxia in the bottom seawater truly occurred in the SNB. Although we know that hypoxia has deleterious implications for bottom-living species [40], we still know little about how and why a lower DO profile occurred at the bottom of SNB.

### 4.2. Vertical Seawater Stratification May Result in Lower DO and Higher Concentrations of Chl-a in SNB

An established theory that explains why lower DO, or in extreme cases called “hypoxia,” occurs in coastal bottom waters involves enhanced riverine NO_3_^−^-N or P loading [2]. This theory states that an increasing amount of nutrient loading promotes the growth of phytoplankton, which produce abundant organic material [41]. Following the sinking of organic material to the bottom seawater, its decomposition will consume O_2_ [42]. When vertical seawater stratification occurs in summer, significant concomitant decreases in O_2_ in the bottom layer may occur [43]. In our work, a significantly lower DO profile in the bottom seawater was detected in the SNB. Considering that the concentration of Chl-a in SNB was higher than that in the DFR in September, we speculated that this theory explains the decrease in the DO in the bottom layer of SNB. However, relatively higher levels of inorganic NO_3_^−^-N or P were not detected in SNB, as we expected. Therefore, high NO_3_^−^-N- or P-driven growth of phytoplankton did not truly exist in SNB.

To explain why lower DO and higher concentrations of Chl-a appeared in SNB, we believe that high-temperature-induced thermal stratification (HTITS) of seawater in the SNB in summer should be taken into account. Thermal stratification results in decreased mixing depth in the oceans or lakes [44]. Due to the decreased mixing depth, the temperature of surface seawater is higher, while the temperature of seawater in the middle layer is lower (below 30 °C) [45]. Undoubtedly, suitable temperatures are beneficial for phytoplankton to survive, reproduce optimally, and maintain relatively higher biomass [46]. During thermal stratification, surface water contains the lowest concentration of Chl-a, and Chl-a gradually increases to the highest value, after which Chl-a decreases at the bottom [47], and phytoplankton density shows a similar variation trend as that in Chl-a. Near the SNB region, the construction of the SDH and the artificial island significantly changed the hydrodynamic conditions [12], subsequently resulting in thermal stratification of seawater in September [13], followed by phytoplankton blooms. In the DFR region, despite higher levels of nutrient N and P loading from anthropogenic activities than those in the SNB region, the intrusion of freshwater from the Dafeng River completely mixes the seawater. Subsequently, the seawater from the surface to the bottom is homogeneously distributed, and the temperature is uniform (totally above 30 °C). This temperature is not suitable for the growth of most kinds of phytoplankton. Therefore, compared to that in SNB, the concentration of Chl-a in the DFR was significantly lower.

### 4.3. Lower DO in Bottom Seawater Significantly Alters Functional Bacterial Groups

FAPROTAX can be used for a fast-functional screening or grouping of 16S-derived bacterial data from ecosystems [35]. Thermal stratification and lower dissolved oxygen in bottom seawater may have significantly altered the functional bacterial groups near SNB in September. The abundance of functional microbial groups involved in anoxygenic photoautotrophy, sulfate respiration, sulfur compound respiration, and anoxygenic photoautotrophic S-oxidization was significantly higher in SNB than in the other areas. The microbiota involved in these metabolic pathways generally live under hypoxic or anoxygenic conditions [48]. Thus, the result is consistent with the fact that a decrease in DO occurred in the bottom layer of SNB in September. Sulfate-reducing bacteria (SRB) are believed to live in an anaerobic environment. The genes *dsrA* and *dsrB* were sensitive gene markers for the quantification of SRB in environmental samples [29,49]. The SRB dominance in SNB initiates a cascade of ecological degradation. SRB reduce SO_4_^2−^ to H_2_S, acidifying sediments (pH < 6.5) and mobilizing heavy metals (e.g., Cd, Pb). H_2_S toxicity causes mass mortality of oxygen-sensitive crustaceans/mollusks [14]. Loss of key prey species forces dolphin foraging shifts to DFR [10].

Highly abundant bacteria that carry out sulfide-driven anoxygenic photosynthesis play an important role in the carbon and nitrogen cycles, as well as the formation and maintenance of the ecosystem. Sulfide-driven anoxygenic photosynthesis contributes significantly to inorganic carbon fixation in stratified, sulfidic water bodies [50]. Moreover, anoxygenic phototrophic bacteria, such as nonsulfur purple bacteria, were reported to conduct nitrogen (N_2_) fixation and reduce N_2_ to NH_3_ [51]. N_2_ fixation is important for maintaining the fertility of nutrient-poor oceans by providing bioavailable nitrogen to support the production of organic matter [52]. Carbon and N_2_ fixation provide additional N and C for ecosystems, which partially explains the higher Chl-a and primary productivity in SNB that occurred in the absence of sufficient terrestrial N or C inputs compared to that in the DFR, in September. The emergence of higher Chl-a and primary productivity in SNB, along with the lower water mobility, further promotes profound changes in the marine ecosystem in the SNB area.

The PLS-PM paths reveal a causal hierarchy in ecosystem degradation: infrastructure-induced stratification (↑temperature, ↓DO) directly enriches anaerobic taxa (e.g., SRB) (Direct pathway). Nitrogen loading (e.g., aquaculture runoff) fuels phytoplankton blooms (↑Chl-a) (Figure S2), whose sinking biomass consumes oxygen via respiration—indirectly expanding anaerobic niches (Indirect pathway). This synergy explains why SNB exhibits severe microbial shifts despite moderate nitrogen inputs: physical drivers (stratification) prime the system for hypoxia, while nitrogen amplifies its effects. The elevated N, Chl-a, and physical factors of seawater may increase the abundance of OTUs involved in anoxygenic photoautotrophy, sulfate respiration, respiration of sulfur compounds, and anoxygenic photoautotrophic S oxidation. Although the nitrogen inputs to the area near SNB are currently lower than those in the DFR estuary region, most likely, the nutrient supply will continuously increase because of continuous loading from river runoff, aquaculture farming, and other anthropogenic activities in the future [53]. The increased nutrient-N may create high-biomass phytoplankton and high Chl-a concentrations in the middle or subsurface seawater where the temperature is comparably lower [54]. When phytoplankton sink to the bottom, the degradation of organic matter consumes a vast amount of O_2_ [42], ultimately significantly impacting the bacterial assemblage in the bottom seawater. Obviously, this may have deleterious implications on the coastal ecosystem near SNB and pose an ecological risk if we do not take any measures to delay its progress in the future.

### 4.4. Environmental Factors Significantly Shape the Microbiota in the Co-Occurrence Network

Environmental factors also significantly shaped the microbiota in the co-occurrence network. Some OTUs in module DFR included some autotrophic bacteria, such as *Tistlia* [55], *Methylotenera* [56], *Sulfuriferula* [57], and *Curvibacter* [58]. This result likely illustrated an ecological network in which some autotrophic bacteria lived by sulfur oxidation, nitrogen fixation, and denitrification in the DFR estuary. Moreover, a bacterial predator, *Halobacteriovorax* [59,60], was detected in module DFR. *Halobacteriovorax* may prey on bacteria and release nutrients into seawater [60]. The recycling of nutrients maintains the growth of phytoplankton and heterotrophic bacteria. The OTUs enriched in the network of SNB included some microbial groups, such as *Dinoroseobacter* [61,62], *Seonamhaeicola* [63], and *Seonamhaeicola* [64], which have established a close relationship with phytoplankton. The OTUs in the network enriched in SNB also included some bacteria closely related to some species involved in the denitrification of nitrate (*Paracoccus*) and nitrogen fixation (*Propionispira*) [65]. Therefore, the co-occurrence network revealed two functionally distinct microbial guilds: The SNB module, with *Dinoroseobacter* (algal symbiotic bacteria) and *Paracoccus* (denitrifying bacteria) as the core, it forms an “algae-degrader alliance” (Module II, accounting for 42% of OTUs), which promotes organic matter sedimentation and oxygen consumption; and DFR module, dominated by *Sulfuriferula* (sulfur-oxidizing bacteria) and *Methylotenera* (nitrogen-fixing bacteria), it constitutes a “sulfur cycle-autotrophic network” (Module I, accounting for 38% of OTUs), maintaining nitrogen and sulfur cycles in low-nutrient environments.

**Figure 9:**
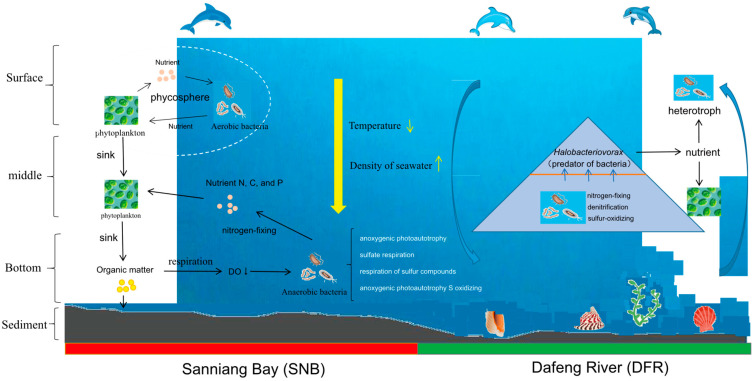
Hypothesized ecological network in Sanniang Bay (SNB) and the Dafeng River (DFR). SNB’s bacterial communities centered on phytoplankton-associated denitrifiers (e.g., Dinoroseobacter), while DFR harbored sulfur oxidizers and nitrogen fixers, indicating distinct biogeochemical pathways. The mutually reciprocal “phytoplankton–bacteria” relationship accelerated the accumulation of organic matter near SNB, and the increase in organic matter and black muddy soil aggravated lower DO conditions in the bottom seawater. Consequently, healthy benthic ecosystem in SNB was destroyed. The yellow ↓ indicate a decrease in value, the yellow ↑ indicate an increase in value.

We speculate that a phytoplankton–bacteria interaction may play an important role in the biogeochemical cycles in the SNB region. First, in this ecosystem, some aerobic bacteria in the phycosphere live by degrading the organic matter secreted by algae. Algae, in turn, utilize nutrients, such as vitamin B12 and nitrogen, secreted by bacteria to survive [66]. Second, the organic particles sink to the bottom layer, and O_2_ consumption during respiration results in a drastic decrease in O_2_ in the bottom seawater. Consequently, the lower dissolved oxygen contributes significantly to the proliferation of bacteria involved in anaerobic metabolism and accelerates the accumulation of nutrients in the ecosystem, which benefits the survival of phytoplankton (Figure 9).

PLS-PM analysis illustrated that environmental factors driving the interaction of bacteria in the ecological network were notably different in SNB and the DFR. This may provide some useful implications about the impact of environmental characteristics on the ecosystem and inform solutions. First, some microbes enriched in SNB in the network were positively impacted by seawater properties, N, C, and P. From the network analysis, we conclude that organic matter, phytoplankton, aerobic bacteria, and anaerobic bacteria have established a stable ecological network. An additional input of nutrients, N or P, will accelerate the accumulation of organic matter and black muddy soil near the SNB region (Figure 9). Furthermore, the increase in organic matter and black muddy soil will aggravate lower DO conditions in the bottom seawater. Consequently, healthy benthic ecosystems will be destroyed. This scenario is likely to occur when terrestrial N and P pollution or aquaculture wastewater is discharged into seawater in this coastal region. Considering that SNB is a well-known distribution area of humpback dolphins in China [16], some strict measures reducing nitrogen and phosphorus emissions should be considered. Second, we should pay more attention to the impact of land reclamation (construction of the SDH) on the health of ecosystems in SNB. Before construction, the SDH was thought to be able to prevent the loading of N, C, and P into SNB and subsequently reduce eutrophication in this region. However, the SDH changed the hydrodynamic conditions and resulted in the summer stratification of seawater, changing the seawater properties, consequently impacting the ecological network (just as the PLS-PM analysis illustrates in Figure 8), finally resulting in a series of deleterious consequences for the ecosystem. This study provided us with a novel and representative example of the impact of land reclamation on the ecosystem.

### 4.5. Ecological Implications of Lower DO for Benthic Fauna and Fisheries

The observed DO decline (5.44 and 7.09 mg L^−1^) approached sub-optimal state (4.0–4.8 mg L^−1^) but did not reach the defined hypoxia threshold (<2 mg L^−1^). While technically classified as “decreased DO” rather than hypoxia, these levels are ecologically critical, as prolonged exposure near the hypoxia threshold can induce chronic stress in benthic organisms [39]. Benthic organisms such as crustaceans (e.g., shrimp, crabs) and mollusks (e.g., clams, oysters) are highly sensitive to hypoxia [67,68], as demonstrated by their significant reduction in SNB following SDH construction [14]. These species play critical roles in sediment bioturbation, enhancing nutrient exchange and oxygen penetration [69]. Their decline disrupts sediment stability, leading to diminished activity of burrowing organisms and resulting in compacted, anoxic sediments, further inhibiting aerobic microbial processes and promoting sulfide accumulation [70]. Hypoxia-tolerant taxa (e.g., polychaetes) may dominate, but their low nutritional value cascades up the food chain, affecting predators like demersal fish [2]. The collapse of benthic prey directly impacts commercially valuable fish species, which rely on crustaceans and mollusks as primary food sources. Hypoxia-induced mortality of benthic invertebrates reduces forage availability, forcing fish to migrate or face starvation. Fish avoid hypoxic zones, compressing their distribution into smaller areas (e.g., DFR), increasing competition and susceptibility to overfishing [71]. The decline in high-value species (e.g., shrimp, grouper) has shifted fishing efforts to lower-trophic-level fish, exacerbating overexploitation and reducing profitability. This trend mirrors hypoxia-affected regions like the Gulf of Mexico [71].

### 4.6. Lower DO and Its Ecological Implications for Humpback Dolphins

From this study, we can find the answer to why the utilization of SNB decreased obviously and the core distribution sites shifted to a previously low-disturbance site, the DFR [16]. Hypoxia (DO < 2 mg L^−1^) directly impacts benthic invertebrates (e.g., crustaceans, mollusks) and demersal fish, which constitute the main diet of the humpback dolphin in SNB [16]. Declines in these prey groups have been documented in SNB since SDH construction [14], mirroring hypoxic zones globally where fish avoid low-oxygen “dead zones” [2]. Hypoxia-tolerant species (e.g., jellyfish, polychaetes) often dominate degraded benthic communities, but these are nutritionally inadequate for dolphins [72]. This trophic mismatch may explain reduced dolphin sightings in SNB and increased foraging in the DFR, where freshwater mixing maintains higher DO [10,16]. Hypoxic sediments in SNB host sulfate-reducing bacteria, which produce neurotoxic hydrogen sulfide (H_2_S). Benthic prey exposed to H_2_S and heavy metals (e.g., Cd, Pb detected in SNB sediments) may transfer toxins up the food chain, posing risks of bioaccumulation in dolphins [14]. Chronic exposure to hypoxia and contaminants can elevate stress hormones (e.g., cortisol), weakening immune responses and increasing susceptibility to disease—a pattern documented in bottlenose dolphins in hypoxic estuaries [73].

### 4.7. Evidence-Based Adaptive Management Strategies

Based on mechanistic insights from PLS-PM and microbial indicators, we propose some targeted interventions. Since the SDH infrastructure disrupts hydrodynamic connectivity, we can convert marine highways into bridges to enhance water flow. In order to reduce phytoplankton blooms driven by September TN peaks, we should implement “wet season fertilizer bans” (July–October) in watershed farmlands and constructed wetlands (30% area coverage) to intercept agricultural runoff. Since hypoxic sediments suppress benthic prey [14], we could transplant seagrass (e.g., *Halodule uninervis*) in SNB to enhance sediment oxygenation during peak hypoxia (September–October) [69].

## 5. Conclusions

This study demonstrates that summer thermal stratification (induced by SDH infrastructure) drives pronounced dissolved oxygen decline (5.44 and 7.09 mg L^−1^) in Sanniang Bay (SNB) bottom waters, triggering fundamental shifts in microbial functionality:

(1) Anaerobic dominance: SRB (*dsrA*/*dsrB* abundance: SNB > DFR, *p* < 0.01) and anoxygenic phototrophs increased in SNB, confirming hypoxia adaptation.
(2) Hierarchical drivers: PLS-PM quantified seawater properties as the primary direct driver of anaerobic taxa, with nitrogen loading exerting secondary effects via phytoplankton blooms.
(3) Network divergence: SNB’s co-occurrence network centered on phytoplankton-associated denitrifiers (e.g., Dinoroseobacter), while DFR harbored sulfur oxidizers and nitrogen fixers, indicating distinct biogeochemical pathways.

These findings establish microbial biomarkers (e.g., SRB genes, anoxygenic taxa) as early indicators of incipient hypoxia, providing a mechanistic basis for targeting infrastructure-mediated stratification in coastal management. The PLS-PM model quantifies that managing seawater properties (e.g., via restoring hydrodynamics) is more effective than nitrogen reduction alone in mitigating anaerobic microbiota. This underscores the need to prioritize SDH infrastructure modifications alongside nutrient control.

## Data Availability

The high-throughput sequencing data from this research have been uploaded to GenBank with the BioProject accession numbers SRR23110292—SRR23110307.

## Supplementary Materials

The following supporting information can be downloaded at: https://www.mdpi.com/article/10.3390/microorganisms13081945/s1, Figure S1. The bathymetric data of the sampling sites; Figure S2. Spatial and temporal distributions of environmental variables (NH_4_^+^, NO_3_^−^, Ch-a, temperature, and salinity) in the SNB and DFR regions in the Beibu Gulf. The values of the environmental variables are interpolated on the map using the kriging method. The blue to red colors indicate lower and higher concentrations, respectively. SAL: salinity (‰); TEMP: temperature.
