# Supplementary material for: Dissolved Oxygen Decline in Northern Beibu Gulf Summer Bottom Waters: Reserve Management Insights from Microbiome Analysis
Source: Microorganisms. 2025 Aug 20;13(8):1945. doi: 10.3390/microorganisms13081945 (PMC12388384; doi:10.3390/microorganisms13081945)
Supplement: Supplementary file 1 [file microorganisms-13-01945-s001.zip › microorganisms-3735877-supplementary.pdf]

Dissolved Oxygen Decline in Northern Beibu Gulf Summer Bottom Waters: Reserve Management Insights from Microbiome Analysis

Chunyan Peng †, Ying Liu †, Yuyue Qin, Dan Sun, Jixin Jia, Zongsheng Xie and Bin Gong *

The Guangxi Key Laboratory of Beibu Gulf Marine Biodiversity Conservation, College of Marine Sciences, Beibu Gulf University, Qinzhou 535011, China; pcybbgu@163.com (C.P.); liuying06shengke@163.com (Y.L.); qyy12021221@163.com (Y.Q.); sundan0991@163.com (D.S.); jiajixin521@gmail.com (J.J.); xzs249832@163.com (Z.X.)

* Correspondence: gongbin@bbgu.edu.cn

† These authors contribute equally to this work.

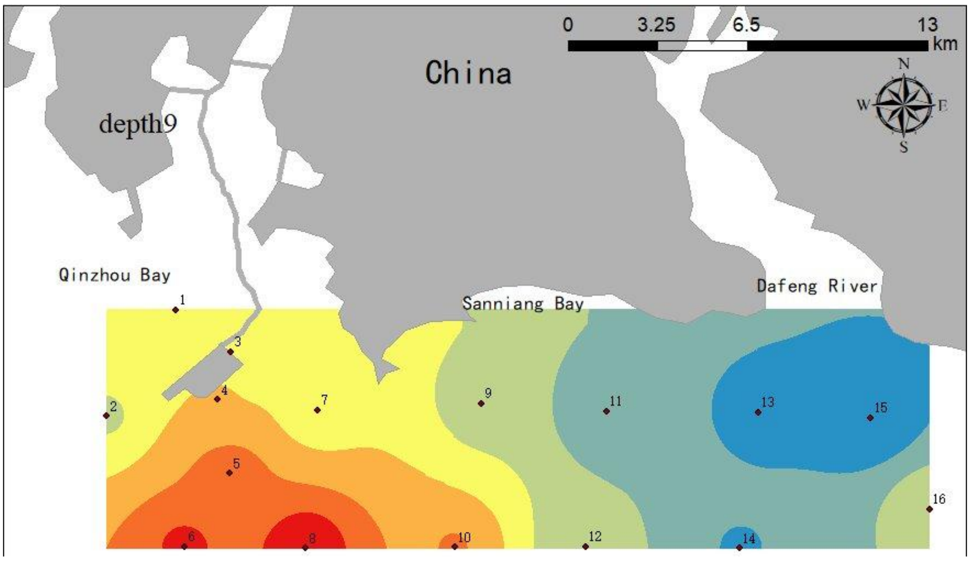

Figure S1. The bathymetric data of the sampling sites. The depth of 16 sampling stations (S1-S16) ranged from 2.3-10.7 m. The colors, ranging from "blue→yellow→red", indicate the depth variation from 2.3 meters to 10.7 meters (Blue represents shallow areas, and red represents deep areas).

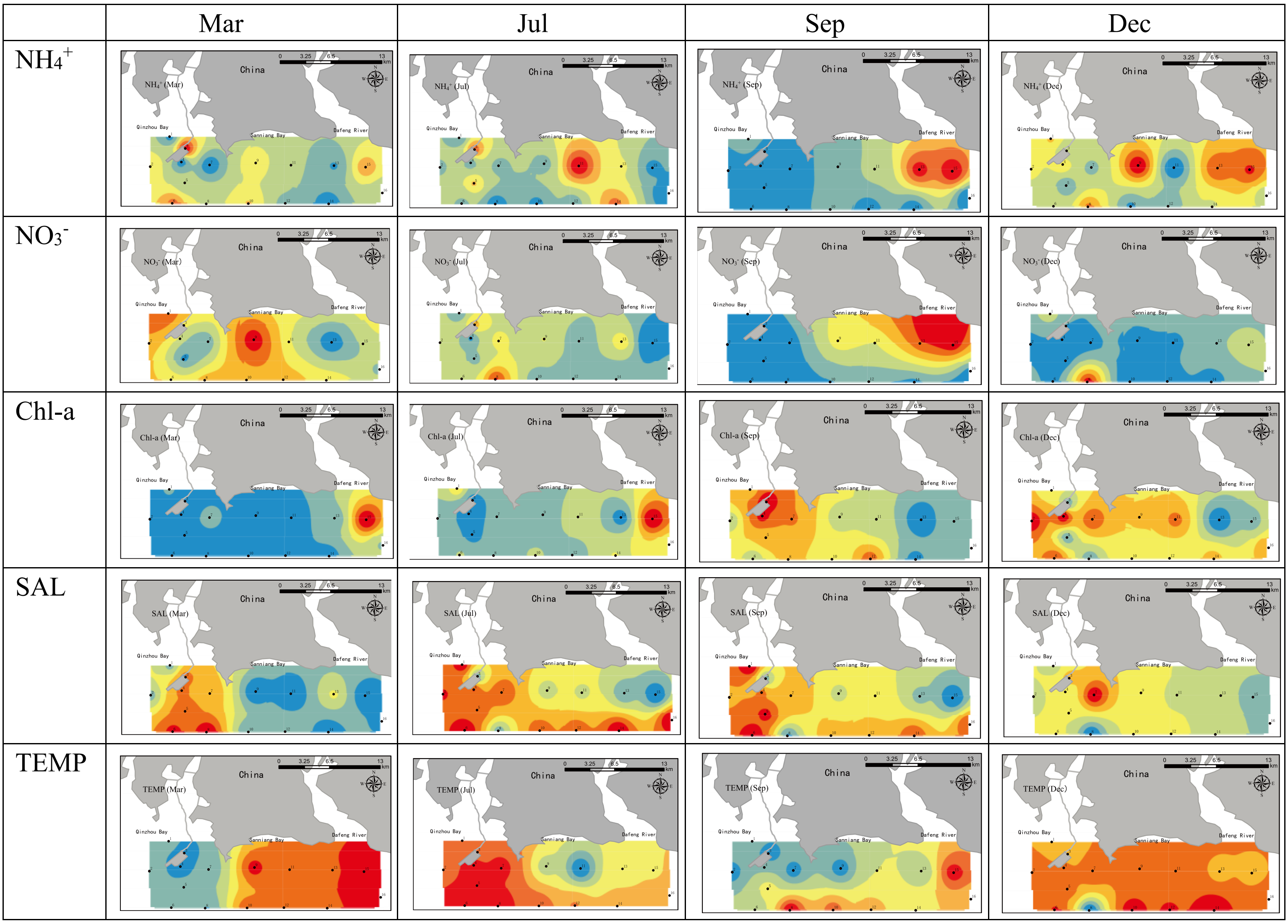

Figure S2. Spatial and temporal distributions of environmental variables (NH_4^+, NO_3^-, Ch-a, temperature, and salinity) in the SNB and DFR regions in the Beibu Gulf. The values of the environmental variables are interpolated on the map using the kriging method. The blue to red colors indicate lower and higher concentrations, respectively. SAL: salinity (‰); TEMP: temperature.
